# Supplementary figures and images for: Development and validation of an interpretable machine learning model for predicting left atrial thrombus or spontaneous echo contrast in non-valvular atrial fibrillation patients
Source: PLoS One. 2025 Jan 16;20(1):e0313562. doi: 10.1371/journal.pone.0313562 (PMC11737704; doi:10.1371/journal.pone.0313562)

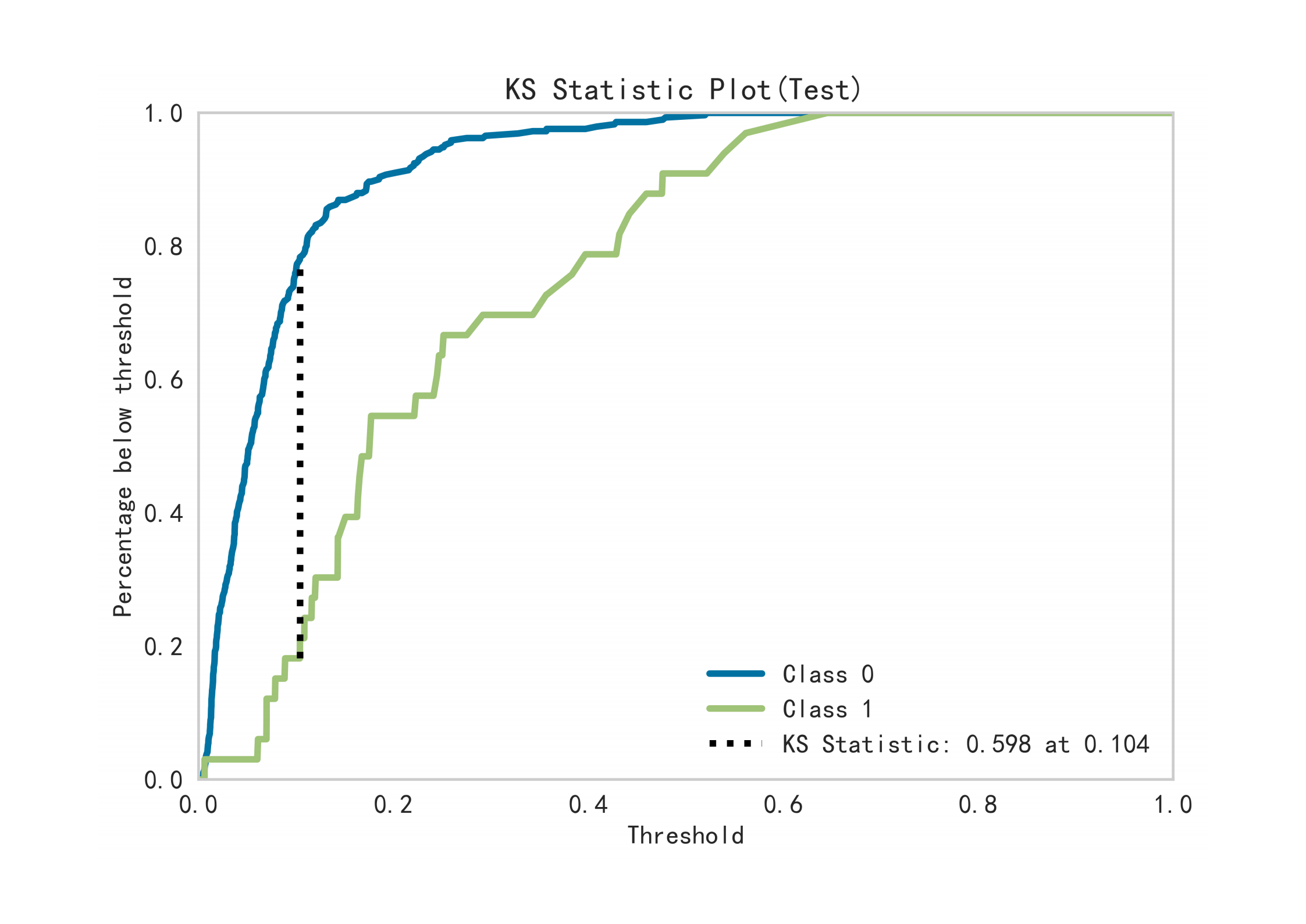

Supplement: S1 Fig — (TIF) [file pone.0313562.s001.tif]

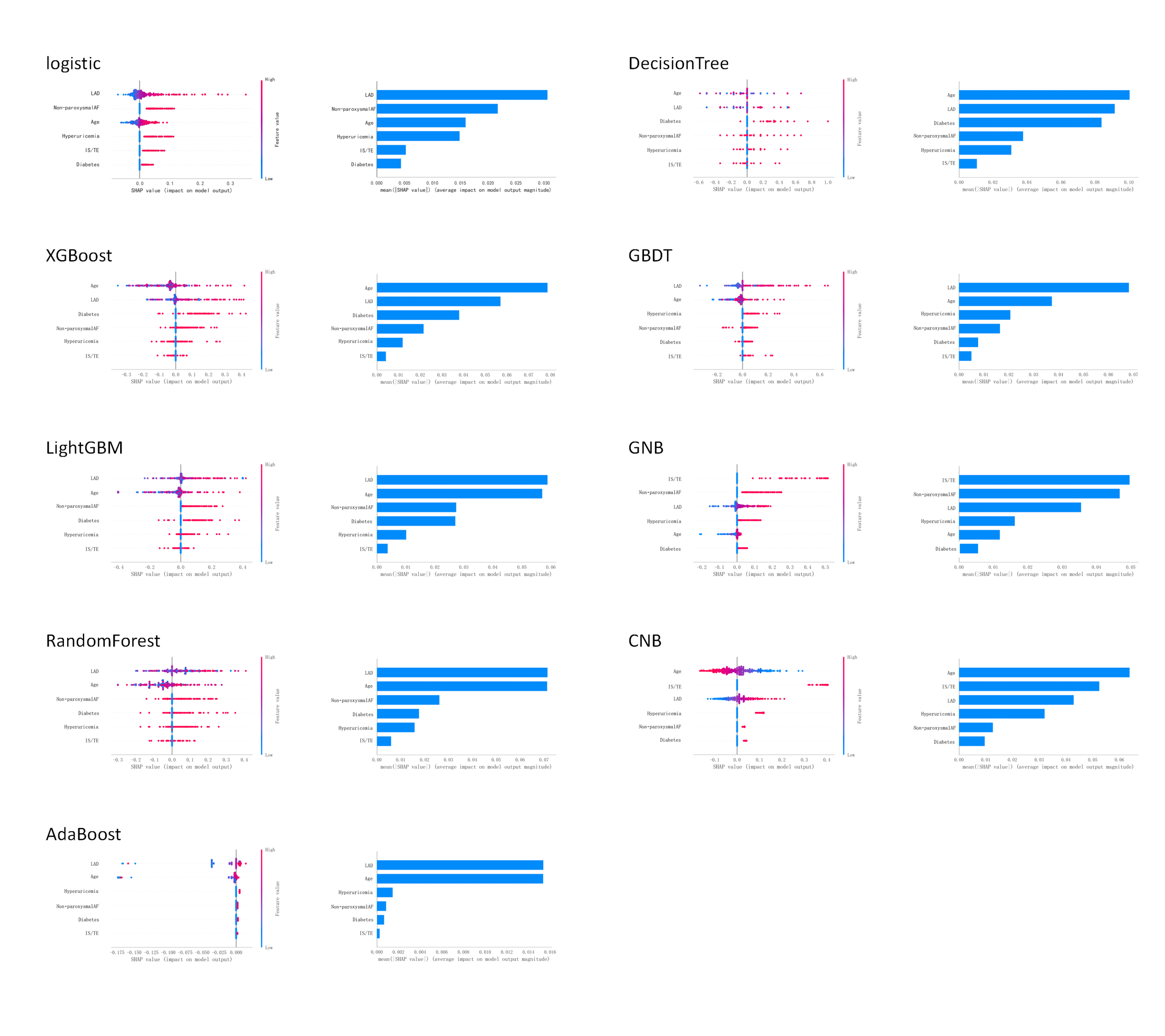

Supplement: S2 Fig — (TIF) [file pone.0313562.s002.tif]
